# Novel Endometrial Cancer Models Using Sensitive Metastasis Tracing for CXCR4-Targeted Therapy in Advanced Disease

**DOI:** 10.3390/biomedicines10071680

**Published:** 2022-07-12

**Authors:** Esperanza Medina-Gutiérrez, María Virtudes Céspedes, Alberto Gallardo, Elisa Rioja-Blanco, Miquel Àngel Pavón, Laura Asensio-Puig, Lourdes Farré, Lorena Alba-Castellón, Ugutz Unzueta, Antonio Villaverde, Esther Vázquez, Isolda Casanova, Ramon Mangues

**Affiliations:** 1 Institut d’Investigació Biomèdica Sant Pau (IIB-Sant Pau), 08041 Barcelona, Spain; emedina@santpau.cat (E.M.-G.); mcespedes@santpau.cat (M.V.C.); erioja@santpau.cat (E.R.-B.); lalba@santpau.cat (L.A.-C.); uunzueta@santpau.cat (U.U.); 2 Institut de Recerca contra la Leucèmia Josep Carreras, 08025 Barcelona, Spain; 3 Department of Pathology, Hospital de la Santa Creu i Sant Pau, 08041 Barcelona, Spain; agallardoa@santpau.cat; 4 Institut Català d’Oncologia (ICO), 08908 L’Hospitalet de Llobregat, Spain; mpavon@iconcologia.net (M.À.P.); lfarre@iconcologia.net (L.F.); 5 CIBER en Epidemiología y Salud Pública (CIBERESP), 28029 Madrid, Spain; 6 Institut d’Investigació Biomèdica de Bellvitge (IDIBELL), 08908 L’Hospitalet de Llobregat, Spain; 7 Program Against Cancer Therapeutic Resistance (ProCURE), Catalan Institute of Oncology (ICO), Oncobell Program, Bellvitge Biomedical Research Institute (IDIBELL), 08908 L’Hospitalet del Llobregat, Spain; lasensio@idibell.cat; 8 CIBER de Bioingeniería, Biomateriales y Nanomedicina (CIBER-BBN), 28029 Madrid, Spain; antoni.villaverde@uab.cat; 9 Departament de Genètica i de Microbiologia, Universitat Autònoma de Barcelona, 08193 Bellaterra, Spain; 10 Institut de Biotecnologia i de Biomedicina, Universitat Autònoma de Barcelona, 08193 Bellaterra, Spain

**Keywords:** advanced endometrial cancer, orthotopic model, metastasis, CXCR4-targeted nanoparticles, animal model

## Abstract

Advanced endometrial cancer (EC) lacks therapy, thus, there is a need for novel treatment targets. CXCR4 overexpression is associated with a poor prognosis in several cancers, whereas its inhibition prevents metastases. We assessed CXCR4 expression in EC in women by using IHC. Orthotopic models were generated with transendometrial implantation of CXCR4-transduced EC cells. After in vitro evaluation of the CXCR4-targeted T22-GFP-H6 nanocarrier, subcutaneous EC models were used to study its uptake in tumor and normal organs. Of the women, 91% overexpressed CXCR4, making them candidates for CXCR4-targeted therapies. Thus, we developed CXCR4^+^ EC mouse models to improve metastagenesis compared to current models and to use them to develop novel CXCR4-targeted therapies for unresponsive EC. It showed enhanced dissemination, especially in the lungs and liver, and displayed 100% metastasis penetrance at all clinically relevant sites with anti-hVimentin IHC, improving detection sensitivity. Regarding the CXCR4-targeted nanocarrier, 60% accumulated in the SC tumor; therefore, selectively targeting CXCR4^+^ cancer cells, without toxicity in non-tumor organs. Our CXCR4^+^ EC models will allow testing of novel CXCR4-targeted drugs and development of nanomedicines derived from T22-GFP-H6 to deliver drugs to CXCR4^+^ cells in advanced EC. This novel approach provides a therapeutic option for women with metastatic, high risk or recurrent EC that have a dismal prognosis and lack effective therapies.

## 1. Introduction

Endometrial cancer (EC) is the most common cancer of the female genital tract, and the sixth most diagnosed cancer in women [1,2]. Approximately 3–14% of EC patients are younger than 40 and want to spare their fertility [3]. The standard treatment for low grade EC is surgery, with prior fertility-sparing treatments when required [4,5]. Still, advanced stages or high-risk EC patients currently lack effective therapies. Even though EC has a five-year survival rate of 80% [6], it decreases dramatically to a 56% or 20% rate when locoregional or distal metastasis occurs [7,8]. This dismal prognosis is due to the inability of cisplatin/paclitaxel chemotherapy or radiotherapy to effectively block metastatic dissemination [9].

In this regard, there is a need to develop animal models that mimic the metastatic progression and pathological features found in EC in humans, which could improve the knowledge of the molecular drivers of EC metastases in different organs and allow the adequate testing of drugs that may block dissemination during their preclinical development [10]. Nevertheless, among the available orthotopic preclinical models only a few replicate the advanced stages of EC, and when they do, they still show important limitations for testing novel drugs. Thus, some models do not reach a 100% engraftment to ensure the generation of primary tumor in all implanted mice and/or display high interindividual variability in metastatic foci development at clinically relevant sites [11,12,13,14,15]. However, most reported metastatic EC models are heterothopic since the location of EC cells implantation is other than the endometrium. Therefore, they are unable to mimic the primary tumor microenvironment and are scarcely relevant in the investigation of the possible molecular or cellular pathways that drive EC metastases in humans [16,17,18,19,20,21,22].

An additional and highly relevant issue among the published disseminated EC models, is the low sensitivity of most of the techniques used to detect metastatic foci at the different sites. Most researchers detect metastases identifying foci by using optical microscopy in tissue sections after hematoxylin-eosin (H&E) staining, whereas others generate cell-line-derived bioluminescent models that allow in vivo and ex vivo EC tracking. Nevertheless, neither method can reliably detect single cells or small cell clusters that infiltrate the organs where metastases are expected. This low sensitivity leads to the loss of relevant information on tumor cell arrival and colonization of distal organs. Therefore, there is a need to improve the metastatic cell detection threshold to unequivocally spot tumor cells that infiltrate distant organs at the single cell level.

The CXCR4 chemokine receptor is overexpressed at mRNA and protein levels in EC, compared to hyperplasia and a normal endometrium, being proposed to play a role in tumor progression in EC [23]. In fact, the CXCR4/CXCL12 pathway has been associated with EC progression [24,25]. This is consistent with reports demonstrating that overexpression of this receptor is associated with poor prognosis and/or enhanced metastases in at least 20 cancers, both solid and hematological [26,27]. In EC, there are inconclusive works regarding CXCR4 expression as a possible prognostic factor [28,29], similar to findings in breast cancer, in which CXCR4 expression shows a different prognosis depending on the tumor subtype [30,31]. Therefore, the role of CXCR4 in EC is still controversial regarding its possible effect on tumor growth or metastatic dissemination [27,32,33]. Interestingly, in subcutaneous EC models, CXCR4 expression increases EC cell engraftment and tumor growth rate [24,34,35,36]. However, to date, the study of the effect of CXCR4 overexpression on metastatic dissemination in EC mouse models has not been addressed yet, whereas in a majority of evaluated cancers, CXCR4 overexpression is associated with a worse prognosis [26].

Here, we generated a new CXCR4-overexpressing (CXCR4^+^) orthotopic EC mouse model, using a novel surgical procedure, and set up a highly sensitive immunohistochemical tumor cell marker to detect small metastatic foci and single cancer cells in clinically relevant organs. Using this methodology, we have demonstrated that CXCR4 overexpression enhances metastatic dissemination in EC. Next, we developed a CXCR4^+^ subcutaneous EC model to be used to demonstrate a highly specific accumulation of the CXCR4-targeted nanocarrier T22-GFP-H6 in EC. We had previously produced this nanocarrier [37] and also reported high tumor uptake in cancer models other than EC [38,39,40]. Thus, these novel EC models could be a new resource for developing CXCR4-targeted therapies that provide a non-surgical therapeutic option for women who lack effective therapies, such as those in advanced stages or high-risk EC patients.

## 2. Materials and Methods

### 2.1. Endometrial Cancer Patient Samples

Patient samples were collected under the *Hospital de la Santa Creu i de Sant Pau* Ethics Committee, with informed consent. It included a retrospective collection of 102 archived paraffined tissue samples, including normal and tumor tissue, from 79 women with endometrial cancer. Cylindrical tissue cores of 3 mm diameter were extracted from different paraffin blocks and re-embedded into single recipients (microarrays), to perform CXCR4 immunohistochemistry.

### 2.2. Cell Culture

#### 2.2.1. Cell Line Constructs and Stable Cell Line Generation

AN3CA cell line (ATCC, Manassas, VA, USA) was cultured in DMEM and F12 medium 1:1; HEC1A cell line (ATCC, USA) and ARK-2 (kindly provided by Dr. Matias-Guiu) were cultured in high glucose DMEM medium. All of them were maintained in medium supplemented with 10% fetal bovine serum and penicillin/streptomycin, at 37 °C and 5% CO_2_. Cells were tested for *Mycoplasma* sp. contamination using LookOut Mycoplasma PCR Detection Kit (Sigma-Aldrich, Taufkirchen, Germany) once every three months.

#### 2.2.2. Lentiviral Transduction

For CXCR4 and/or luciferase expression, AN3CA was transduced with either pLentiIII-UbC-CXCR4-Luciferase or pLentiIII-UbC-Luciferase plasmids using lentivirus. After 48 h, cells were selected with puromycin (1 μg/mL for AN3CA, 3 μg/mL for HEC1A and 5 μg/mL for ARK-2).

#### 2.2.3. Flow Cytometry

##### Membrane CXCR4 Assessment

CXCR4 membrane expression was determined using anti-CXCR4 antibody (PE-Cy5 mouse anti-human CD184, BD Biosciences, Franklin Lakes, NJ, USA) and its control (PE-Cy5 mouse IgG2a, K isotype control, BD), by flow cytometry in FACScalibur (BD Biosciences).

To obtain homogeneous cell populations, with high intensity of CXCR4 in the membrane in a large percent of the population, cells were sorted using FACSAria (BD Biosciences). 

##### Internalization Assay

1 mL of a suspension of 500,000 CXCR4^+^ Luciferase^+^ AN3CA cells/mL was seeded in 6 well plates for 24 h. Then, they were exposed to T22-GFP-H6, a protein-based nanocarrier that targets CXCR4, at different concentrations (1, 10, 50, 100. 400 nM) for 1, 6 or 24 h. The competition assay for CXCR4-dependent internalization was performed by preincubating the cells with 1 µM of the CXCR4 antagonist AMD3100 for 1 h before exposure to T22-GFP-H6.

Quantification of nanocarrier internalization in cells (GFP^+^ cells) was performed using flow cytometry. After cell detachment with trypsin, cells were washed with PBS, and treated with trypsin-EDTA (1 mg/mL, Life Technologies, Carlsbad, CA, USA) to remove nonspecific binding of T22-GFP-H6 binding to cell membrane.

Results were analyzed with software CellQuest Pro and expressed as percentage of fluorescent cells or mean fluorescence intensity.

#### 2.2.4. Cell Viability Assay

100 µL 250,000 CXCR4^+^ AN3CA cells/mL per well were seeded in 96 well plates. Then, 24 h later, they were incubated with T22-GF-H6 in concentrations ranging from 10 nM to 400 nM. The competition assay was performed by preincubating the cells with 1 µM CXCR4 antagonist AMD3100 for 1 h. Cell viability was assessed using Cell Proliferation Kit II (XTT, Roche, Basel, Switzerland). Absorbance at 490 nm was read 48 h later (FLUOstar OPTIMA, BMG Labtech, Ortenberg, Germany). Data were shown as percentage of viable cells as compared to the viability of buffer-exposed cells.

#### 2.2.5. Cell Blocks

Cells were seeded in 150 cm^2^ cell culture flasks. Once they reached a 70–80% confluence, cells were washed, trypsinized, collected and washed in PBS. After 5 min centrifugation at 400× *g*, cell blocks were obtained mixing gently 2 drops of human plasma and 2 drops of human thrombin. Cell pellets were fixed in paraformaldehyde 4% and embedded in paraffin to further CXCR4 analysis by immunocytochemistry.

### 2.3. In Vivo Experiments

Five-week-old female Swiss nude (Crl:NU(Ico)-*Foxn1^nu^*) or NSG (NOD.Cg-*Prkdc^scid^ Il2rg^tm1Wjl^/SzJ*) mice (Charles River, France) were used to develop in vivo subcutaneous and orthotopic models, respectively. After an acclimatization period of 7 days, they were housed in groups of five, in individually ventilated cage units (15.40 × 7.83 × 6.30 inch, Sealsafe Plus GM500, Techniplast, West Chester, PA, USA), with individual poplar chips bedding and its cellulose bag as environmental enrichment (Sodispan). Mice were maintained under specific pathogen-free conditions and a light/dark cycle of 12 h. They were fed with irradiated food (14% protein, 4% fat, Teklad Global diet, ENVIGO, Indianapolis, IN, USA) and reverse osmosis autoclaved water ad libitum. During surgery, eye hydration was maintained with a saline drip, and hypothermia was avoided using a heating blanket under a drape to keep body temperature at 37 °C. All the animal procedures were performed using sterile material in a type II biosafety cabinet (BIO-II-A/P, Telstar, Barcelona, Spain).

Randomizations of animals in experimental groups were performed using dice. The investigator was not blinded to group allocation during in vivo manipulation of animals; nevertheless, they were blinded for the histological analysis.

All mice were euthanized by cervical dislocation once they arrived at the experimental endpoint or reached endpoint criteria (10–20% body weight loss, signs of pain or distress such as abnormal postures, ulcers, alopecia, ruffled fur, abnormal breathing, abnormal activity, coma, ataxia, tremors). All the in vivo procedures were approved by the *Hospital de la Santa Creu i de Sant Pau* Animal Ethics Committee and Generalitat de Catalunya (FUE-2018-00819447, 24 April 2019), and performed according to European Council directives (CEA-OH/9721/2).

#### 2.3.1. Orthotopic EC Models

NSG mice were anesthetized with 100 mg/kg ketamine (Ketolar, Pfizer, New york, NY, USA) and 10 mg/kg xylazine (Rompun, Bayer, Leverkusen, Germany). The lower abdomen was shaved and swabbed with povidone. A medial laparotomy was performed to expose the right uterine horn. Its irrigation system was separated, and a ligature was performed in the proximal section of the horn using 7/0 Optilene (BBraun, Melsungen, Germany).

After randomization (*n* = 4/group), either 10^6^ CXCR4^+^ AN3CA or CXCR4^-^ AN3CA cells resuspended in 25 ul of culture medium were inoculated through the myometrium into the endometrial cavity using a 29G Hamilton syringe (Microliter Serie 800, Hamilton, Reno, NV, USA).

Overall animal health conditions were monitored three times a week. All mice were left to survival, and they were sacrificed as soon as they reached human endpoint criteria (55 ± 17 days; mean ± SD). In addition to the previously stated endpoint criteria, for this experiment, high primary tumor or peritoneal carcinomatosis growth (determined by palpation or a maximum whole body bioluminiscence of 2 × 10^10^ p/s/cm^2^/sr) as well as abdominal distension, were observed.

#### 2.3.2. Subcutaneous CXCR4^+^ EC Models

Swiss nude mice were anesthetized with 2% isoflurane, and their backs were swabbed with povidone-iodine. In both flanks, 10^7^ CXCR4^+^ AN3CA cells were inoculated subcutaneously (SC). Overall animal health and tumor growth were monitored twice a week, using a caliper and applying the formula V = width^2^ × length/2. For the setup experiment (*n* = 4), endpoint was set at 600 mm^3^, which was reached 15–30 days after inoculation.

#### 2.3.3. In Vivo T22-GFP-H6 Biodistribution

Nanocarrier biodistribution was assessed in the SC EC mouse model following the experimental design shown in Figure S1. A total of 19 animals with subcutaneous tumor development were randomized in control (*n* = 3) or treated groups (*n* = 4) for different time points (2, 5, 24 and 48 h) once tumors reached 150–200 mm^3^. All of them received intravenously 200 µL of buffer or 200 µg T22-GFP-H6, respectively. Fluorescence intensity emitted by the GFP domain of T22-GFP-H6 was measured ex vivo, after euthanasia at 2, 5 or 24 h in SC tumors and non-tumor organs (liver, kidney, lung, spleen). Emitted fluorescence intensity was expressed as the average radiant efficiency [(p/s/cm^2^/str)/(mW/cm^2^)] since this reflects the accumulation of T22-GFP-H6 in each tissue, once the autofluorescence of control mouse tissues is subtracted.

#### 2.3.4. Necropsy and Histological Examination

After necropsy and ex vivo assessment of biodistribution or bioluminescence emission, tumor and non-tumor organs were collected and fixed in 4% formaldehyde for histopathological or immunohistochemical evaluation, during which the investigator was blinded for the animal group.

All organs were stained with hematoxylin-eosin (H&E) to evaluate tumor histologic architecture (stromal, vascular development or possible necrosis) and possible toxicity or nanocarrier accumulation.

### 2.4. Bioluminescence Intensity Assessment

Luciferase expression in vitro was assessed by seeding 500,000 cells per well on 6 well plates. Bioluminescence emission was expressed as radiance photons (p/s/cm^2^/sr) and registered using IVIS Spectrum 200 equipment, adding 200 μL D-luciferin firefly potassium salt 1.0 g (15 mg/mL, Perkin Elmer, Waltham, MA, USA). Images were analyzed using Living Image v.4.7.3. software. 

To assess the correct implantation of cells in vivo, mice were injected intraperitoneally with firefly d-luciferin (2.25 mg/mouse, Perkin Elmer), which is a substrate for luciferase which is expressed in CXCR4^+^ EC cells. Mice were anesthetized with 3% isoflurane in oxygen to capture bioluminescence intensity 5 min after injection. Images were analyzed using Living Image v.4.7.3. software.

### 2.5. Immunocytochemistry and Immunohistochemistry

Immunocytochemical (ICC) and immunohistochemical (IHC) staining were performed in a DAKO Autostainer Link48 (Agilent, Santa Clara, CA, USA) following the manufacturer’s instructions, using 4 μm paraffin sections of cell blocks or organs.

The human CXCR4 staining pattern in cells was assessed using anti-CXCR4 (1:200; Abcam, ab124824) in all cell blocks, patient tissue samples and tumor and non-tumor organs. After assessing human vimentin (V9, Dako, IGA63061-2) expression in subcutaneous tumors, it was used to determine the presence or absence of tumor cells in non-tumor tissues. Ki67 (Dako GA62661-2) was used to determine the proliferation profile of subcutaneous tumors.

All slides were examined by a blind observer under an optic microscope and representative pictures were taken using an Olympus DP73 camera. Liver sections were processed with cellSens software (Olympus, RRID:SCR_014551, Tokyo, Japan), while lung sections were analyzed with QuPath [41], both at 200× magnification.

### 2.6. Statistical Analysis

In vitro experiments (internalization, XTT) were performed in biological triplicates. Differences between groups were analyzed using the Mann–Whitney test, prior determination of normality by the Shapiro–Wilk test using SPSS software v.23. Differences were considered statistically significant at *p* ≤ 0.05. In vivo experiments (mice models setup, nanocarrier biodistribution) were performed in quadruplicates, defined by previous experiments regarding the interindividual variability among mice in terms of tumor growth and metastatic load. No animal exclusion was done. Randomization was performed using dice; odd numbers were assigned to group 1, while even numbers were assigned to group 2. Cohen’s delta was performed to assess the size of differences in lung and liver metastatic load between animal groups, using R software v.3.4.4. The effect size was considered large when *d* > 0.8 and medium when *d* > 0.5 [42,43]. All results were expressed as the mean ± standard error (s.e.m.).

## 3. Results

### 3.1. Immunohistochemical Evaluation of CXCR4 Expression in EC Patient Samples

**Figure 1:**
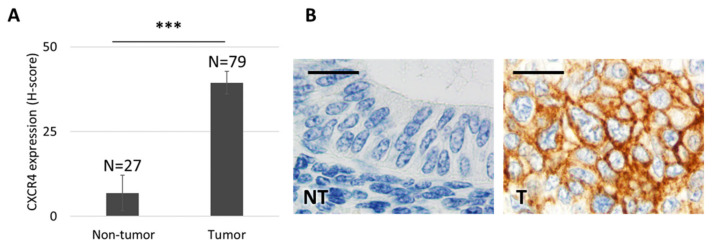
CXCR4 expression in tissue microarrays obtained from endometrial cancer patients. (**A**) CXCR4 is overexpressed in tumor tissue (*n* = 79), as compared to adjacent healthy endometrial tissue (*n* = 27), that shows almost undetectable CXCR4 expression levels by immunohistochemistry (IHC) (Mann–Whitney test, *** *p* = 0.000; mean ± s.e.m). (**B**) CXCR4 protein expression after IHC is negligible in healthy endometrial tissue, whereas it is highly overexpressed and mostly located in the cell membrane of tumor endometrial tissue. Bar: 20 µm. NT: non-tumor, healthy tissue; T: tumor tissue.

To assess CXCR4 expression levels and the localization pattern in EC tumors, we performed an IHC staining of this protein using tissue samples from 79 patients diagnosed with EC at *Hospital de la Santa Creu i Sant Pau*. CXCR4 was highly overexpressed in 91.6% of tumor tissue, as compared to the level displayed by healthy cells in endometrial tissue. Moreover, a majority of EC patients (64.4%) overexpressed CXCR4 in their membrane (Figure 1). Patients with membrane CXCR4 overexpression become candidates for targeting of CXCR4^+^ EC cells, including selective drug delivery, through receptor-mediated internalization, as a therapeutic strategy. 

### 3.2. Generation of CXCR4^+^ Luciferase^+^ Human EC Cell Lines

We evaluated CXCR4 expression levels in two endometrioid EC human cell lines, AN3CA and HEC1A, and one serous EC human cell line, ARK2. All three showed negligible levels of CXCR4 as measured both by flow cytometry and immunocytochemistry (ICC) (Figure S2). Therefore, they were transduced with CXCR4-Luciferase lentiviral vectors.

All transduced EC cell lines, including AN3CA, ARK2 and HEC1, became CXCR4^+^ Luciferase^+^ and therefore displayed high bioluminescent emission (Figure S3A). However, the highest expression of membrane CXCR4 was achieved by the bioluminescent AN3CA cell line (Figure S2B). In this sense, while the transduced, luminescent AN3CA cell line maintained high CXCR4 expression in the membrane, as seen by flow cytometry and ICC, transduced HEC1A cells showed a high CXCR4 expression. However, according to CXCR4 ICC, its expression pattern was mostly cytoplasmatic. Similarly, ARK-2 showed an increase in CXCR4 levels when transduced, but these levels did not remain intense or steady over passages. Thus, the CXCR4^+^ Luciferase^+^ AN3CA cell line was the only one that maintained membrane CXCR4 overexpression over time, therefore being selected for further in vitro and in vivo work.

### 3.3. Development of a Subcutaneous Tumor Model Bearing Human EC Cells in Swiss Nude and Follow-Up Markers of Cancer Cell Growth 

We developed a CXCR4^+^ subcutaneous model, implanting 10^7^ CXCR4^+^ Luciferase^+^ AN3CA cells in the flank of Swiss nude mice to validate tumor cells engraftment and growth (Figure S3B). We obtained a 100% engraftment rate, and luciferase emission was assessed over time. The bioluminescence emitted by the SC tumor correlated with tumor size, even though there was a certain variability in tumor growth rate among mice (Figure S4A).

After necropsy, tumor histology was evaluated by H&E, showing mostly EC epithelial cells jointly with stromal and vascular tissue areas with no signs of necrosis neither macroscopically nor microscopically (Figure S4B). Tumors also showed high Ki67 expression that indicated high proliferative activity and also a high membrane CXCR4 levels in cancer epithelial cells as we had already observed in vitro. Moreover, the human mesodermal marker vimentin was expressed, showing a highly intense staining in in EC epithelial cells, and a lack of staining in all mouse cells, including stroma and endothelium (Figure S4B).

### 3.4. Bioluminescent Follow-Up of Primary Tumor and Metastatic Dissemination in a Novel Orthotopic Model of Advanced EC in NSG Mice

**Figure 2:**
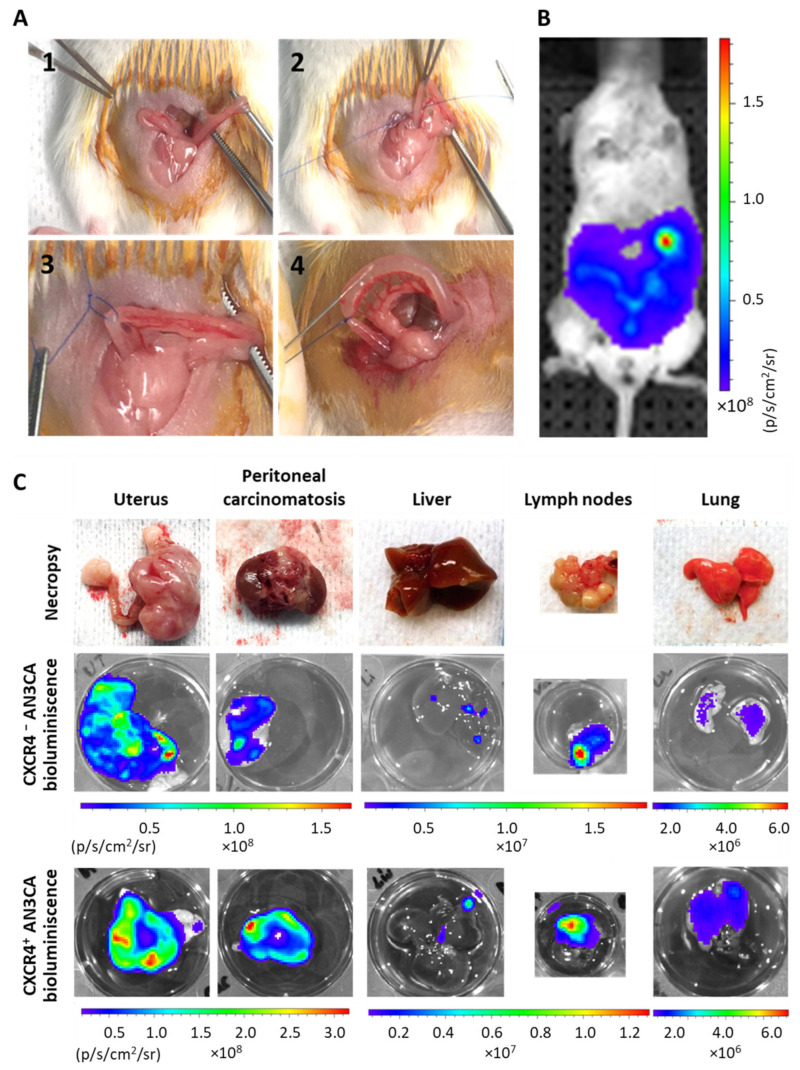
Procedure to orthotopically inoculate human cell lines in NSG mice to obtain an aggressive endometrial cancer model that metastasizes in all clinically relevant organs. (**A**) Representative photographs of the taken procedure, starting by showing uterus exposure (1), followed by right horn ligature without clamping the arterial irrigation system (2 and 3) and intraluminal transmyometrial injection of 10^6^ CXCR4^+^ Luciferase^+^ AN3CA or CXCR4^-^ Luciferase^+^ AN3CA cells suspended on culture medium using a Hamilton syringe. (**B**) Representative image of Luciferase bioluminiscence emission in a mouse imlpanted with Luciferase^+^ AN3CA cells, indicating the correct engraftment of tumor cells inside the uterus. (**C**) Representative images of relevant organs bearing metastases after necropsy and their ex vivo emission of bioluminiscence, registered by IVIS Spectrum.

We developed a unique procedure to generate a novel orthotopic mouse model of endometrial cancer by ligation of the horn and transmyometrial injection of CXCR4^+^ Luciferase^+^ or CXCR4^-^ AN3CA cells (Figure 2). The ligature avoided vaginal leakage of the injected cell suspension (Figure 2A). Bioluminiscence allowed us to know whether local or distant tissues were colonized by EC cells. Nevertheless, a histological analysis or an IHC evaluation yielded a more precise identification and quantitation of the disseminated EC cells, as reported in Figure 2. Thus, bioluminescence proved to be useful in identifying the presence of EC cells in distant organs (Figure 2B), despite being only a qualitative marker, showing that our model replicates the sites of metastases observed in advanced EC patients. This fact entails an improvement over previously reported models, because of its take rate of 100% of the inoculated mice in all clinically relevant organs, including the uterus (primary tumor) and ovaries, the peritoneal cavity, lymph nodes, and the liver and lungs (Figure 2C).

### 3.5. Marker-Guided Comparison of Metastatic Yield in the EC Intrauterine Orthotopic Models Generated from CXCR4^-^ or CXCR4^+^ EC Cells in NSG Mice

**Figure 3:**
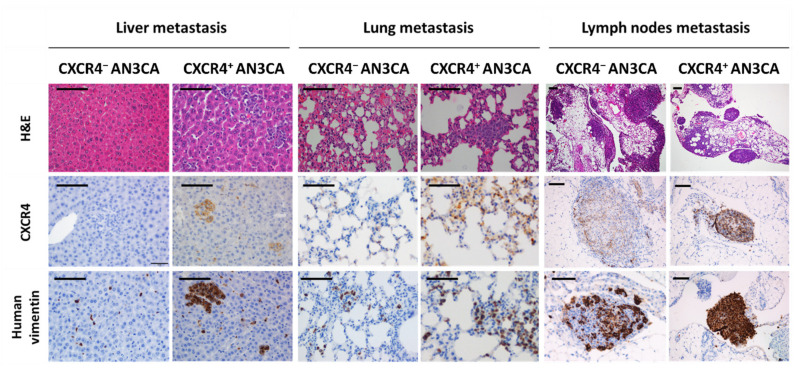
Dissemination pattern of the xenograft orthotopic model of advanced endometrial cancer generated in NSG mice and comparison of dissemination between models derived from CXCR4^+^ and CXCR4^−^AN3CA cells. Histology of liver, lungs and lymph nodes with possible metastatic colonization after hematoxilin-eosin (H&E) staining of tissue sections or using immunohistochemical staining for anti-human CXCR4 or anti-human vimentin. Note the dramatically higher sensitivity of the anti-human vimentin IHC staining, to spot single or clustered cancer cells in the mouse tissues as compared to anti-CXCR4 IHC or H&E-stained tissues. Bar: 100 µm.

Anti-human CXCR4 or anti-human vimentin antibodies were shown to be specific and highly sensitive markers in tumor cells in our CXCR4^+^ model. Nevertheless, the mesodermal marker for human vimentin allowed the most sensitive and precise staining for the detection of microscopic metastases, when compared with CXCR4 IHC staining or H&E staining (Figure 3). Anti-human vimentin provides an easier and highly reliable quantification of foci number and their area in paraffined tissue slides, that can be standardized using image analysis software such as Image-J, cellSens or QuPath.

The high sensitivity of human vimentin staining allows the comparison of the metastatic dissemination pattern and the metastatic load, as single cell foci invading the tissue or cell clusters, generated in the orthotopic model derived from CXCR4^+^ AN3CA cells with those generated by the orthotopic model derived from CXCR4^-^ AN3CA cells (Figure 3). In fact, when comparing the metastases in clinically relevant sites such as the liver, lungs and lymph nodes, after IHC staining with anti-human vimentin, we observed that the total liver foci number was higher (*d* = 0.92) in the CXCR4^+^ AN3CA model than in the CXCR4^-^ AN3CA model, particularly in clustered cells foci (*d* = 2.3). Similarly, the total area occupied by the metastatic foci in the lung was higher in the CXCR4^+^ AN3CA model than in the CXCR4^-^ AN3CA model (*d* = 1.26) (Table 1). Therefore, CXCR4 overexpression was substantively associated with a more metastatic and aggressive phenotype in the ORT model than the one achieved with CXCR4^-^ AN3CA cells.

### 3.6. In Vitro Uptake of the Fluorescent T22-GFP-H6 Nanocarrier and Its Cytotoxicity in Human EC Cell Lines

**Figure 4:**
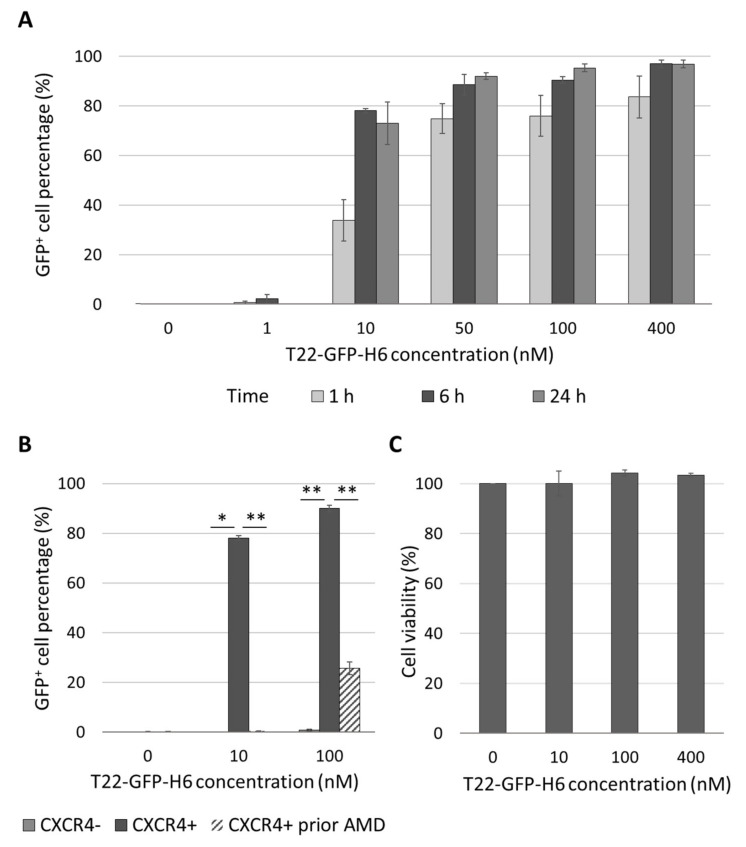
In vitro CXCR4-dependent internalization and lack of cytotoxicity of T22-GFP-H6 nanocarrier in human endometrial cancer cell line AN3CA. (**A**) T22-GFP-H6 internalization in CXCR4^+^ AN3CA cells at 1, 6 and 24 h and different concentrations, expressed as percentage of GFP^+^ cells. (**B**) Blockage of T22-GFP-H6 internalization at 6 h, measured by flow cytometry, in CXCR4^-^ AN3CA cells, CXCR4^+^ AN3CA cells and CXCR4^+^ AN3CA cells after 1h pretreatment with 1 µM of the CXCR4 antagonist AMD3100 (Mann-Whitney test; * *p* < 0.05, ** *p* < 0.01; *n* = 3; mean ± s.e.m). (**C**) T22-GFP-H6 cytotoxicity on CXCR4^+^ AN3CA cells as measured by XTT viability test at 48 h. (*n* = 3; mean ± s.e.m).

Once we determined that CXCR4-overexpression increases the metastatic load in the ORT EC model derived from the AN3CA EC cell line, we wanted to know if the T22-GFP-H6 protein-based nanocarrier could selectively internalize in CXCR4^+^ EC cells. This nanocarrier was previously developed in our group [37], and it targets the CXCR4 receptor, and therefore the CXCR4-overexpressing cancer cells (Figure S5). We observed that the T22-GFP-H6 showed a time and concentration-dependent internalization in the transduced EC cell line CXCR4^+^ Luciferase^+^ AN3CA (Figure 4A). It achieved a high internalization efficiency, as 80% of cells were GFP^+^ after 6 h of exposure to 10 nM T22-GFP-H6. It also showed CXCR4-dependent internalization, as parental CXCR4^-^ cells showed no nanocarrier internalization. Similarly, when CXCR4^+^ cells were preincubated with the CXCR4 antagonist AMD3100 1 h prior to nanocarrier exposure (100 nM T22-GFP-H6), internalization was significantly reduced, while completely blocked at lower concentrations (10 nM T22-GFP-H6) (Figure 4B). Internalization of the nanocarrier in the CXCR4^+^ Luciferase^+^ AN3CA cell line measured as mean fluorescent intensity (MFI) (Figure S6) showed similar levels of T22-GFP-H6 uptake and also demonstrated competition with AMD3100 as described above.

Furthermore, T22-GFP-H6 showed no cytotoxic effect on the CXCR4^+^ AN3CA cell line, as measured by the XTT viability test (Figure 4C). Thus, AN3CA is a good candidate to develop CXCR4^+^ mice models to test the in vivo effect of CXCR4-targeted nanoparticles.

### 3.7. Biodistribution of CXCR4-Targeted Nanocarrier T22-GFP-H6 in a CXCR4^+^ Subcutaneous EC Model

We evaluated the biodistribution of the T22-GFP-H6 nanocarrier in the CXCR4^+^ AN3CA subcutaneous model, measuring its uptake in tumor tissue and in non-tumor organs. For this purpose, we measured the GFP fluorescence emitted along time in the different organs after a single bolus of 200 µg T22-GFP-H6, administered intravenously and compared to the control, as previously described [39].

**Figure 5:**
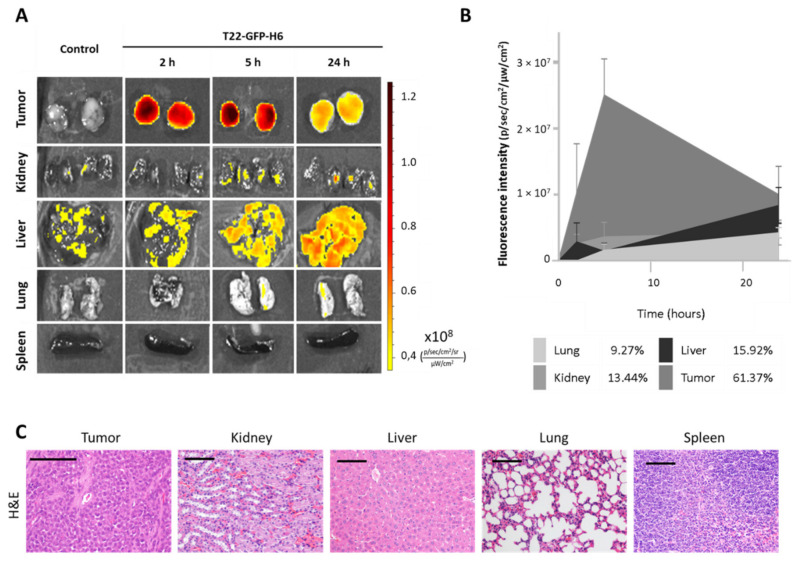
In vivo biodistribution and toxicity assessment of nanocarrier T22-GFP-H6 in a subcutaneous mouse model derived from CXCR4+ AN3CA cells. (**A**) Representative images of fluorescence emitted by the nanocarrier after 2, 5 and 24 h after intravenous injection of 200 µg of T22-GFP-H6. (**B**) Area under the curve representation of fluorescence emitted over time by tumor and non-tumor tissues, and their respective percentage of nanocarrier uptake out of the total fluorescence emitted by T22-GFP-H6 in all tissues (*n* = 4/group; mean ± s.e.m). (**C**) Hematoxylin-eosin staining of tumor and non-tumor organs 48 h after administration of the nanocarrier. Bar: 100 µm.

The nanocarrier was uptaken by the subcutaneous tumor starting 2 h after injection, showing a fluorescence peak at 5 h, which decreased by 24 h (Figure 5A). The total amount of T22-GFP-H6 accumulated in tumor tissue, as measured by the area under the curve of fluorescence intensity over time, was over 60% of the administered dose (Figure 5B). In contrast, H&E staining showed no signs of cell death or histological alteration in tumor tissue at neither of those time points (2, 5 and 24 h) nor at 48 h (Figure 5C).

Non-target organs such as the kidneys, liver, lungs and spleen, also showed normal tissue architecture (Figure 5C) and low (9–16% range) nanocarrier accumulation, as measured by fluorescence emission (Figure 5A,B).

Thus, our nanoparticle has been proven to selectively biodistribute selectively to tumors, in absence of toxicity in tumor or in normal organs.

## 4. Discussion

Aiming to offer a therapeutic option for the treatment of endometrial cancer (EC) patients currently lacking an effective therapy [9], we demonstrated that 64.4% of EC patients overexpress high membrane CXCR4 expression, therefore making them candidates for CXCR4-targeted therapies. Thus, we generated a highly metastatic model to demonstrate that CXCR4 enhances EC metastases, becoming a useful resource for developing CXCR4-targeted therapies against high risk or advanced CXCR4^+^ EC. This novel orthotopic (ORT) CXCR4^+^ EC model, derived from the CXCR4^+^ Luciferase^+^ EC AN3CA cell line, was set up using a new surgical procedure that yields full metastatic penetrance. In addition, we have used an anti-human vimentin antibody to detect metastatic foci, proving this method to be highly sensitive, enabling us to detect even single metastatic cells. Using this model, we have demonstrated that CXCR4-overexpression enhances EC metastatic dissemination to the lungs and liver. Moreover, we have also developed a CXCR4^+^ subcutaneous (SC) model which we next used to demonstrate that the protein-based T22-GFP-H6 nanocarrier that targets CXCR4^+^ cells, displays a high selectivity in its accumulation in CXCR4^+^ EC tumor tissues. The generated EC ORT and SC models can be used for the preclinical testing of novel drug delivery approaches that target CXCR4^+^ EC cells for the treatment of high risk or advanced CXCR4^+^ EC, as well as to study the mechanisms of tumor growth and metastatic dissemination that are dependent on CXCR4 overexpression signaling. Following, we are describing the improvement introduced by our findings, compared to previously published EC models.

### 4.1. CXCR4 Expression Pattern in EC Patients

First, the IHC evaluation of a series of 79 EC patients showed a high CXCR4 receptor expression in 91.6% of primary tumors, as compared to the low level detected in non-tumor endometrium. Remarkably, predominant membrane expression of CXCR4 was detected in 64.4% of patients. Nevertheless, no metastatic orthotopic endometrial cancer model has been described to overexpress it yet, to our knowledge. This reveals a gap in treatment research for EC patients, since a large proportion of them could be candidates for treatments targeted to CXCR4, given its high membrane expression. We have taken advantage of this relevant clinical feature to develop novel CXCR4^+^ EC models, validating its usefulness using a targeted drug delivery approach based in nanoparticles. 

### 4.2. Development of an Aggressive CXCR4^+^ Advanced EC Metastatic Model

The reason for the selection of the AN3CA cell line to generate the advanced metastatic EC model was mainly its high membrane CXCR4 overexpression when transduced, which more closely replicates its expression pattern in patients, as compared to HEC1A or ARK-2 cell lines. Membrane expression of this receptor drives CXCR4-dependent metastatic progression in other tumor types [26], while its role was still unknown in EC. All tested cell lines also expressed luciferase to allow in vivo and ex vivo assessment of primary tumor engraftment and metastatic dissemination follow-up.

The generation for this ORT EC model in immunosuppressed NSG mice applies a novel and easy procedure that consists of the ligation of the horn of the uterus before the transmyometrial injection of the human EC cells. We have produced this model that mimics the highly aggressive behavior observed in advanced EC in humans. Moreover, when CXCR4^+^ AN3CA cells were inoculated, all primary tumor and metastasis maintained the high CXCR4 membrane expression observed in culture. This can be related to the fact that the AN3CA cell line derives from a lymph node EC metastasis, displays high microsatellite instability, *TP53* mutations and *PTEN* deletions and shows resistance to cisplatin and paclitaxel [36,44], all features that are associated with EC aggressiveness [45,46] and relapse [10]. Consistently, the assessment of primary tumors histology showed high grade undifferentiated tumors, with loss of uterine tissue architecture, that clearly resembles cancer progression in patients.

In addition, this novel CXCR4^+^ ORT EC model develops metastases in all clinically relevant sites with 100% penetrance, involving ovaries, abdominal lymph nodes, the peritoneum, liver and lungs, as identified by luminescence emission. Our results clearly improve the models previously described as advanced or metastatic, because they did not accurately replicate the dissemination pattern observed in humans since they were generated through EC cell implantation at heterotopic sites (non-ORT implantation). This kind of cell implantation does not mimic the EC tumor microenvironment [10], yielding, therefore, low rates of tumor engraftment or metastases development, which in turn, limits or precludes the study of the mechanisms of EC metastatic dissemination.

### 4.3. CXCR4 Overexpression Is Associated with Enhanced Metastatic Dissemination in EC

We have also shown that a high level of CXCR4 overexpression in the EC cell membrane (CXCR4^+^) is substantively associated with an increase in metastasis development in the lungs and liver, by comparing the metastatic load between the AN3CA derived CXCR4^+^ and CXCR4^-^ ORT models. This is the first demonstration of the capacity of the overexpression of this receptor to enhance the spread of metastases in EC, which is consistent with previous reports describing the role of CXCR4 on migration in vitro [24,35] and with CXCR4 receptor being overexpressed at mRNA and protein levels in EC, compared to hyperplasia and normal endometrium (Buchynska et al., 2021; Liu et al., 2016; Sun et al., 2017). It is also consistent with the proposed role of CXCR4 in EC progression (Buchynska et al., 2021; Liu et al., 2016) [23,25,36].

### 4.4. Use of Highly Sensitive Human-Vimentin as EC Tumor Cells Marker to Detect Metastatic Foci

Recording the luminescence emitted by cancer cells can spot metastatic foci at the different sites, only when they are large enough and close to the surface of the measured tissue section. Thus, this method is rather qualitative as a tracker of EC foci because of the low penetration of light in tissues.

In contrast, using the immunohistochemical (IHC) detection of human vimentin in tissue sections allowed the easy, reliable and highly sensitive quantification of EC cells in all clinically relevant metastatic organs. Vimentin detection has been previously used as a marker to identify epithelial cancer cells in primary EC tumors [12,15,22], but, to our knowledge, this is the first time that human vimentin is used to detect and quantify the number and area of EC metastatic foci. In this regard, it represents a huge increase in sensitivity as compared to IHC detection of metastases using anti-human CXCR4 or H&E staining. Thus, human vimentin reliably identifies all human tumor cells in the studied section of affected organs, including single EC cells infiltrating the tissue (which anti-CXCR4 or H&E cannot) and small or large size metastatic foci.

The large advantage for human vimentin in scoring metastatic foci could be related to the high affinity of the antibody used for its reaction with the human vimentin epitope. This avoids background staining since it shows no cross reaction with mouse vimentin in tumor stroma or blood vessels, and also because vimentin is expressed on all transformed EC epithelial cells since the endometrium derives from the mesoderm layer in the embryo [47]. This approach dramatically improves the methods used to identify metastatic foci in previously reported metastatic EC models because of the low sensitivity of the used marker or staining (H&E) [12,13,15,17,18,20,22].

This novel application of human vimentin IHC staining could be used, jointly with the detection of molecular drivers of EC dissemination, to investigate the effect of drugs on metastatic colonization, regarding single or cluster cell arrival to the tissue, organ colonization or foci growth at different metastatic sites. Image software for quantitating single or multiple foci in a region of interest or in the whole tissue section will make it even easier to reach this goal. 

### 4.5. Development of a CXCR4 Subcutaneous Tumor Model and Its Use to Evaluate Targeting of Protein-Based Nanocarriers to CXCR4^+^ EC Cells

With the final aim of developing an effective antitumor and antimetastatic drug against this cancer, we started to explore the selectivity of the biodistribution to the tumor tissue and the possible toxicity of the protein-based nanocarrier T22-GFP-H6, which was previously developed by our group and targets CXCR4 [37]. To that purpose, it was first necessary to demonstrate in vitro that T22-GFP-H6 internalization in CXCR4^+^ EC cells was exclusively dependent on CXCR4. Secondly, we developed a CXCR4^+^ SC EC model to evaluate the percent of the administered dose that reaches the tumor tissue, since the subcutaneous tumor model is more suited than the orthotopic model for this specific purpose.

Thus, we performed in vitro experiments that showed that T22-GFP-H6 reached a highly selective, concentration-dependent and CXCR4-mediated internalization in CXCR4^+^ cells. Consistently, this nanocarrier does not internalize in CXCR4^-^ cells; moreover, pretreating CXCR4^+^ cells with the CXCR4 antagonist AMD3100 showed an effective blockage of T22-GFP-H6 internalization. In addition, the nanocarrier displayed neither antitumor activity in EC nor cytotoxicity in normal cells in vitro.

To validate these results in vivo, we used the CXCR4^+^ bioluminescent AN3CA EC cell line to develop a SC EC model with high CXCR4 membrane expression. It produced a 100% rate of tumor engraftment and induced aggressive tumor growth since most of the transformed EC epithelial cells were cycling as demonstrated by Ki67 expression in most cells. It also showed high membrane CXCR4 expression, which has been reported to increase proliferation in SC EC models derived from other cell lines [24,34,35,36]. Moreover, we found a strong correlation between tumor luminescent emission and tumor growth rate.

We used this model to demonstrate that a single intravenous bolus of T22-GFP-H6 leads to its selective accumulation in CXCR4^+^ EC tumors. Thus, 60% of the total emitted fluorescence, out of the total administered dose, is registered in tumor tissue, indicating its CXCR4-dependent internalization. In contrast, its fluorescence level registered in normal organs/tissues was much lower despite showing detectable fluorescence above background (liver, kidney, lung or spleen), while no histological alterations were detected in these or other normal organs.

On this basis, we believe that T22-GFP-H6 nanocarrier is suitable for the development of nanomedicine-based targeted drug delivery approaches for EC therapy since it is not cytotoxic and is highly selective towards CXCR4^+^ EC cells, both in vitro and in vivo. We are now planning to load the nanocarrier with one or a combination of drugs, expecting to achieve a highly selective and sustained delivery of the drug/drugs specifically to tumor tissue with low or negligible drug delivery in normal tissues. This is supported by the high uptake of the nanocarrier by tumor tissue, which starts at 2 h; it lasts until 24 h, and has a peak at 5 h, reaching its accumulation in tumors at much higher levels than those achieved in non-tumor organs. Additional support for its development as a nanomedicine for CXCR4^+^ EC comes from our previous work reporting a similar biodistribution of T22-GFP-H6 in other solid tumors or hematological cancers [38,39,40,48].

### 4.6. Future Contribution of the Novel Models for the Development of Targeted Therapies in Advanced EC

The developed CXCR4^+^ SC EC tumor model could be used to evaluate the antitumor activity of novel CXCR4-targeted drugs, since SC tumor models are widely used in preclinical drug development for EC therapy [10]. However, they do not mimic the microenvironment where EC develops, nor can they be used to assess the effect of drugs on metastatic dissemination. In contrast, the novel ORT CXCR4^+^ EC model developed here ensures a correct implantation of cancer cells in the endometrium, avoiding their leakage, while maintaining the tumor microenvironment where EC develops. It also overexpresses the CXCR4 receptor in their membrane, being able to help in the preclinical development of antimetastatic drugs that target the CXCR4 receptor. Although CXCR4 inhibitors are in clinical trials for other diseases, they have not been tested in EC yet [49]. Our model, being able to assess the antimetastatic effect as well as to possibly predict drug responses to drugs that target this receptor, could have a clinical translation for the treatment of advanced CXCR4^+^ EC in patients. It could also permit us to study the impact of the tested drug on the different processes activated by CXCR4 overexpression during metastasis development (e.g., myometrial or lymphovascular invasion, or colonization of different organs), as well as the evaluation of their underlying mechanisms, measuring their effect on the signaling pathways driving them, since they may closely mimic the clinicopathological features, and the molecular and cellular mechanisms observed in humans with CXCR4-overexpressing advanced EC. 

In addition to its orthotopic implantation, the metastatic model above improves previous metastatic models reported by other authors since it achieves 100% metastasis penetrance in all relevant organs. Moreover, to our knowledge, we report for the first time the quantification of the number and area of metastatic foci, or the total area occupied by these foci in different organs using a highly sensitive cancer cell marker in an ORT EC model, as compared to the mere description of the presence or absence of metastases. Thus, our approach allows the application of statistical tests, with enough power, to determine if there are, or are not, significant differences in metastatic load among the compared groups. Therefore, we believe that both a high metastatic penetrance and a thorough characterization of the number and size of the developed metastatic foci are essential to perform antimetastatic drug evaluation, an aspect seldomly found in published metastatic EC models. A penetrance lower than 100% cannot reliably ensure that the antimetastatic effect achieved is due to the effect of the tested drug or to the lack of EC engraftment in primary tumor or metastatic sites. In addition, a lack of description regarding the number and size of metastases reduces the ability to identify the possible antimetastatic effect of the tested drug. 

Importantly, the development of the SC CXCR4^+^ EC model allowed us to prove that our previously developed protein nanocarrier shows high accumulation in CXCR4^+^ EC tumors because of its capacity to selectively target the CXCR4 receptor. Based on these results, further development of a nanomedicine loading T22-GFP-H6 with drugs of choice for EC would be fully feasible. Then, our next goal will be to generate antimetastatic nanomedicines that achieve targeted drug delivery, to test them in the novel CXCR4^+^ SC and advanced ORT EC models. We expect these nanomedicines to selectively deliver to EC tissues a payload drug of choice (e. g., genotoxic, microtubule inhibitor, toxin…) with high cytotoxic activity in EC in vitro, expecting first to inhibit tumor growth in the CXCR4^+^ SC EC model, and then, to achieve the control of metastatic dissemination in the CXCR4^+^ ORT EC model. A successful preclinical development will, then, initiate an effort to reach clinical translation for patients in most need of new therapies—medically inoperable patients or those who bear highly metastatic, high risk or recurrent EC—selecting only as candidates for treatment, patients bearing CXCR4^+^ EC, since they are more likely to respond.

## 5. Conclusions

In conclusion, we have developed a CXCR4-overexpresing (CXCR4^+^) advanced EC model that improves previously reported models, regarding its CXCR4 overexpression resembling human tumors, and its metastatic penetrance, which was 100% in all clinically relevant sites. Using highly sensitive IHC to detect human vimentin, we reliably identified single EC cells and EC metastatic clusters invading tissues, improving the precision of the metastatic foci detection in EC, in terms of both foci number and size. In addition, we have used this model and this IHC method to demonstrate for the first time that CXCR4-overexpression enhances metastatic dissemination in EC. This model can be used as a resource for the development of therapeutic approaches that target CXCR4, which is overexpressed in EC, especially aimed at patients who currently lack an effective therapy. This way, CXCR4^+^ EC patients will benefit from CXCR4-targeted therapies, including CXCR4 inhibitors or CXCR4-targeted drug delivery approaches.

## Figures and Tables

**Table 1:** Comparison of metastatic dissemination in advanced endometrial cancer orthotopic models derived from CXCR4^+^ or CXCR4^-^ AN3CA cells.

Inoculated Cell Line	Liver Mets					Lung Mets
	Total Foci		Single Cell Foci	Clustered Cells Foci		Invaded Tissue Area (%)
	Number	Area (µm^2^)	Number	Number	Area (µm^2^)	
**CXCR4^-^ AN3CA**	10.2 ± 6.7 ^a^	615.5 ± 429.5 ^b^	9.4 ± 6.3	0.9 ± 0.5 ^c^	4486.4 ± 2728.0	11.5 ± 4.2 ^d^
**CXCR4^+^ AN3CA**	24.2 ± 8.3 ^a^	2536.8 ± 1746.8 ^b^	15.6 ± 6.5	9.7 ± 2.7 ^c^	5305.0 ± 3517.9	26.1 ± 7.0 ^d^

The xenograft orthotopic models of advanced endometrial cancer were generated in NSG mice by implantation of CXCR4^+^ or CXCR4^-^ AN3CA cells (*n* = 4/group). Results are reported as mean ± s.e.m. of number or area of metastatic foci per mouse counting medium power microscope fields (200×, 10 fields) in liver or lung sections, after IHC staining using anti-human vimentin. Assessment of the effect size between groups was performed using Cohen’s delta (*d*) test. ^a^ *d* = 0.92; ^b^ *d* = 0.75; ^c^ *d* = 2.3; ^d^ *d* = 1.26.

## Data Availability

The datasets generated during and/or analyzed during the current study, as well as additional information and data, are available from the corresponding author upon reasonable request.

## Supplementary Materials

The following supporting information can be downloaded at: https://www.mdpi.com/article/10.3390/biomedicines10071680/s1: Figure S1. Experimental design of the in vivo evaluation of T22-GFP-H6 biodistribution; Figure S2. Comparison of the percentage of CXCR4+ cells with membrane expression after transduction with CXCR4-Luciferase constructs in human endometrial cancer cell lines; Figure S3. Luciferase activity in transduced human endometrial cancer cell lines AN3CA, ARK2 and HEC1A; Figure S4. Tumor growth and phenotypic characterization of the human CXCR4+ AN3CA cell line-derived subcutaneous cancer mouse model; Figure S5. Schematic design and functional versatility of CXCR4-targeted nanocarrier T22-GFP-H6; Figure S6. In vitro CXCR4-dependent internalization of T22-GFP-H6 in CXCR4+ EC AN3CA cells.
