# Supplementary material for: Novel Endometrial Cancer Models Using Sensitive Metastasis Tracing for CXCR4-Targeted Therapy in Advanced Disease
Source: Biomedicines. 2022 Jul 12;10(7):1680. doi: 10.3390/biomedicines10071680 (PMC9313294; doi:10.3390/biomedicines10071680)
Supplement: Supplementary file 1 [file biomedicines-10-01680-s001.zip › biomedicines-1763375-supplementary.pdf]

Supplemental Material

Novel Endometrial Cancer Models Using Sensitive Metastasis Tracing for CXCR4-Targeted Therapy in Advanced Disease

Esperanza Medina-Gutiérrez [1,2], María Virtudes Céspedes [1], Alberto Gallardo [3], Elisa Rioja-Blanco [1,2], Miquel Àngel Pavón [4,5,6], Laura Asensio-Puig [7], Lourdes Farré [4,7], Lorena Alba-Castellón [1,2], Ugutz Unzueta [1,2,8,9], Antonio Villaverde [8,9,10], Esther Vázquez [8,9,10,*], Isolda Casanova [1,2,8,*] and Ramon Mangues [1,2,8,*]

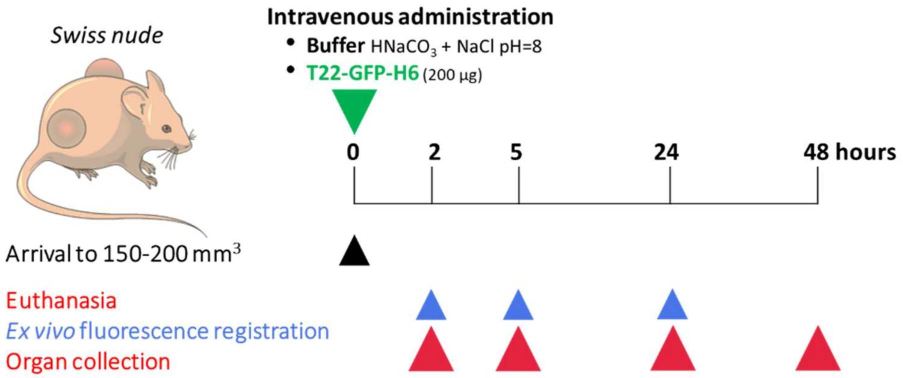

Figure S1. Experimental design of the *in vivo* evaluation of T22-GFP-H6 biodistribution, carried on in the subcutaneous tumor model derived from CXCR4+ AN3CA endometrial cancer cell line in Swiss nude mice. Once the tumor reached 150 to 200 mm3, mice received a single intravenous bolus of either buffer or 200 µg T22-GFP-H6 (n=3-4/group) and were euthanized 2, 5, 24 or 48 hours after the administration.

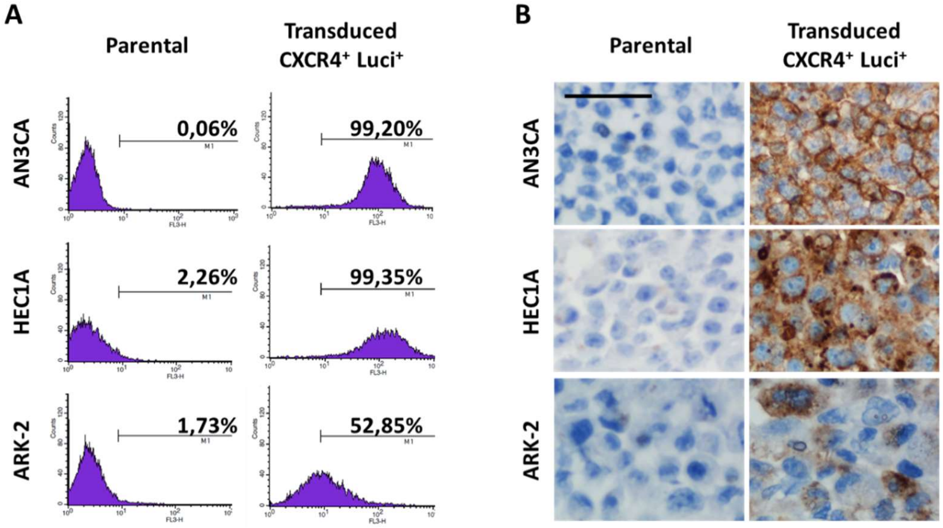

Figure S2. Comparison of the percentage of CXCR4^{+} cells with membrane expression after transduction with CXCR4-Luciferase constructs in human endometrial cancer cell lines. Representative images of CXCR4 expression in parental or lentivirally transduced AN3CA, HEC1A and ARK-2 cell lines, assessed by: **A. Flow cytometry** of suspended cells stained with anti-CXCR4 antibody. **B. CXCR4 immunocytochemistry** staining with anti-CXCR4 antibody on cell pellets. Bar: 50 μm.

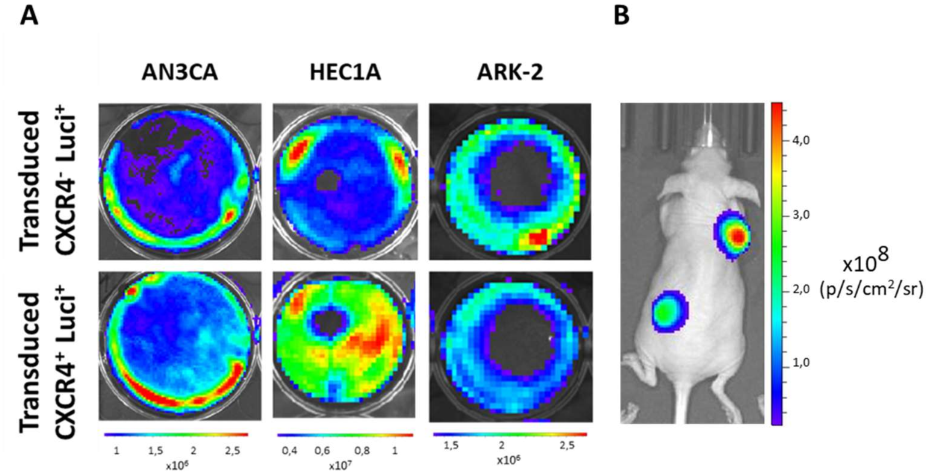

Figure S3. Luciferase activity in transduced human endometrial cancer cell lines AN3CA, ARK2 and HEC1A ***in vitro*** **(A)** and in a **subcutaneous model (B) derived from the implantation of CXCR4^{+} Luciferase^{+} AN3CA cells.**

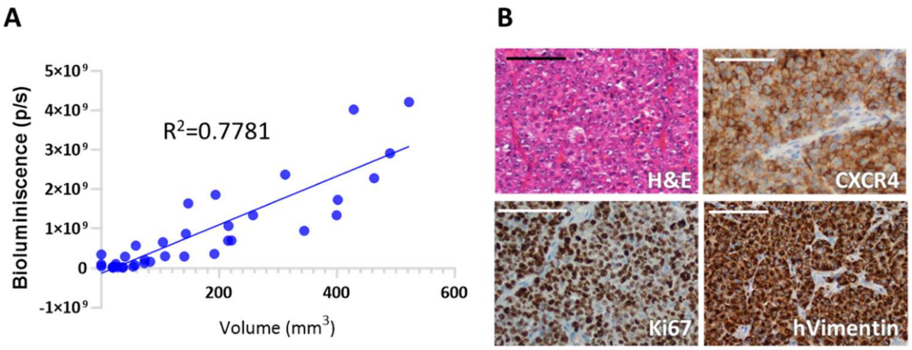

Figure S4. Tumor growth and phenotypic characterization of the human CXCR4⁺ AN3CA cell line-derived subcutaneous cancer mouse model. Swiss nude mice were implanted with 10^7 $CXCR4^+$ $Luciferase^+$ AN3CA cells, to assess bioluminescence emission and tumor growth over time. **A. Correlation between tumor volume and bioluminiscence** emitted by tumor cells (n=8; p<0.0001). **B. Phenotypic histopathologic characterization of tumor tissue** stained with hematoxilin-eosin (H&E) and marker characterization of human cells in a tumor cointaining mouse stroma, using immunohistochemical staining for anti-human CXCR4, Ki67 or human vimentin (hVimentin) antibodies. Bar: 100 µm.

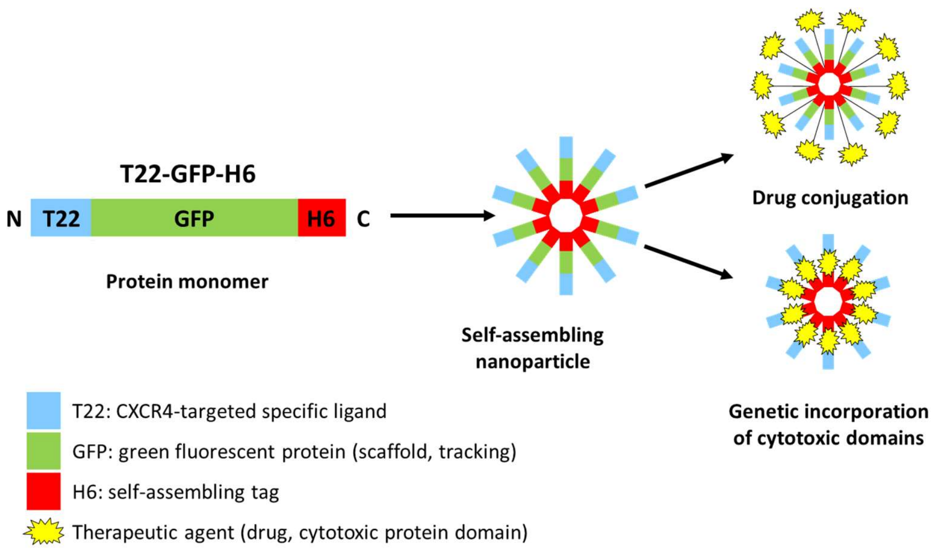

Figure S5. Schematic design and functional versatility of CXCR4-targeted nanocarrier T22-GFP-H6, capable of generating nanomedicines targeting this receptor.

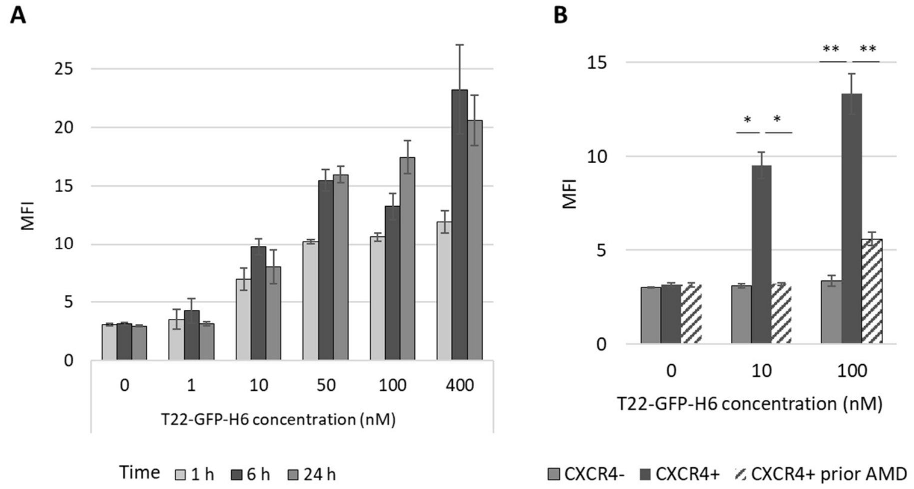

Figure S6. ***In vitro*** **CXCR4-dependent internalization of T22-GFP-H6 in CXCR4⁺ EC AN3CA** cells. A. T22-GFP-H6 internalization in CXCR4⁺ AN3CA cells at 1, 6 and 24 h and different concentrations, expressed as mean fluorescent intensity (MFI). **B. Blockage of T22-GFP-H6 internalization at 6 hours, measured by flow cytometry**, in CXCR4- AN3CA cells, $CXCR4^+$ AN3CA cells and $CXCR4^+$ AN3CA cells after 1h pretreatment with 1 μM of the CXCR4 antagonist AMD3100. T22-GFP-H6 internalization in $CXCR4^+$ cancer cells is expressed as mean fluorescence intensity (MFI). Mean±s.e.m; *p<0.05; ** p<0.01.
